# Examining the level and inequality in health insurance coverage in 36 sub-Saharan African countries

**DOI:** 10.1136/bmjgh-2020-004712

**Published:** 2021-04-26

**Authors:** Edwine Barasa, Jacob Kazungu, Peter Nguhiu, Nirmala Ravishankar

**Affiliations:** 1 Health Economics Research Unit, KEMRI-Wellcome Trust Research Programme, Nairobi, Kenya; 2 Center for Tropical Medicine and Global Health, Nuffield Department of Medicine, University of Oxford, Oxford, UK; 3 Stratetic purchasing for PHC, Thinkwell, Washington, District of Columbia, USA

**Keywords:** health insurance, health systems, health economics

## Abstract

**Introduction:**

Low/middle-income countries (LMICs) in sub-Saharan Africa (SSA) are increasingly turning to public contributory health insurance as a mechanism for removing financial barriers to access and extending financial risk protection to the population. Against this backdrop, we assessed the level and inequality of population coverage of existing health insurance schemes in 36 SSA countries.

**Methods:**

Using secondary data from the most recent Demographic and Health Surveys, we computed mean population coverage for any type of health insurance, and for specific forms of health insurance schemes, by country. We developed concentration curves, computed concentration indices, and rich–poor differences and ratios to examine inequality in health insurance coverage. We decomposed the concentration index using a generalised linear model to examine the contribution of household and individual-level factors to the inequality in health insurance coverage.

**Results:**

Only four countries had coverage levels with any type of health insurance of above 20% (Rwanda—78.7% (95% CI 77.5% to 79.9%), Ghana—58.2% (95% CI 56.2% to 60.1%), Gabon—40.8% (95% CI 38.2% to 43.5%), and Burundi 22.0% (95% CI 20.7% to 23.2%)). Overall, health insurance coverage was low (7.9% (95% CI 7.8% to 7.9%)) and pro-rich; concentration index=0.4 (95% CI 0.3 to 0.4, p<0.001). Exposure to media made the greatest contribution to the pro-rich distribution of health insurance coverage (50.3%), followed by socioeconomic status (44.3%) and the level of education (41.6%).

**Conclusion:**

Coverage of health insurance in SSA is low and pro-rich. The four countries that had health insurance coverage levels greater than 20% were all characterised by substantial funding from tax revenues. The other study countries featured predominantly voluntary mechanisms. In a context of high informality of labour markets, SSA and other LMICs should rethink the role of voluntary contributory health insurance and instead embrace tax funding as a sustainable and feasible mechanism for mobilising resources for the health sector.

**Key questions**

**What is already known?**

- The literature on health insurance coverage in sub-Saharan Africa (SSA) is mostly focused on individual countries and specific forms of health insurance (such as social health insurance or community-based health insurance).
- No study has conducted a multicountry analysis of health insurance coverage with different forms of health insurance in SSA.
- There is also scant literature on inequality in health insurance coverage and certainly no multicountry study that gives a glimpse of inequality in health insurance in SSA.

**What are the new findings?**

- The level of health insurance coverage in SSA is low; only 8 of the 36 countries examined had a mean level of insurance coverage with any type of health insurance of above 10%, while only 4 had a coverage level of above 20%.
- Health insurance coverage in SSA is characterised by substantial income inequalities.
- Exposure to media, socioeconomic rank and the level of education had the greatest contribution to inequality in coverage with any type of health insurance in SSA.

**What do the new findings imply?**

- SSA countries will not achieve Universal Health Coverage using health insurance that is de facto voluntary as a health financing scheme.
- SSA countries should therefore reconsider voluntary contributory health insurance mechanisms and instead reorient their health financing system towards non-contributory tax-funded arrangements.

## Introduction

The inclusion of Universal Health Coverage (UHC) as a health-related Sustainable Development Goal has cemented its position as a global health priority. The goal of UHC is to ensure that everyone has access to high-quality healthcare services that they need, without the risk of financial ruin or impoverishment.^1^ Low/middle-income countries (LMICs) are increasingly prioritising UHC and reforming their health systems to accelerate progress to achieve this target.^2^ To attain UHC, countries need to scale up access to needed services, as well as extend financial risk protection to the whole population.

There is a consensus that financing arrangements featuring prepayment—as opposed to patients paying out of pocket for services at the time of use—are preferable for ensuring financial risk protection.^3^ There are several options for organising prepayment in healthcare financing, with the main categories being a tax-financed government scheme (along the lines of a national health service) and health insurance.^3^ The latter can take the form of social health insurance, private compulsory health insurance and voluntary health insurance (examples include private voluntary health insurance schemes and voluntary community-based health insurance (CBHI)).^3^ A common characteristic of health insurance as a financing scheme is the fact that entitlement to benefits is contributory.^3^ This means that a contribution payment made by or on behalf of the covered individual is required as a condition for access to care under the financing scheme.^3^ The mode of participation in health insurance schemes may be compulsory/mandatory or voluntary.^3^

Historically, a majority of LMICs opted to set up tax-financed government schemes in the mid-to-late 20th century.^4 5^ They were attracted to the potential that such a scheme offers for extending comprehensive coverage to the whole population, raising revenue from a broad base of tax and non-tax sources (as opposed to member contributions), and containing costs through vertical integration. In practice, these schemes have suffered from insufficient and unstable funding, which in turn led many LMICs to introduce user fees in the public sector. Health facilities in the public sector have also come to be associated with inefficiency and low quality of care, which drives households to seek care from private providers and pay out of pocket for it.

Against this backdrop, LMICs are increasingly turning to health insurance as an alternative way of organising prepayment healthcare financing.^6^ While many of these countries had previously set up social health insurance schemes for formal sector employees and witnessed a mushrooming of CBHI schemes in recent decades,^7 8^ they are now embracing the idea of large publicly owned health insurance that extends coverage to all. In sub-Saharan Africa (SSA), Ghana, Kenya, Nigeria, Rwanda and Tanzania have established such schemes, while countries such as Ethiopia, Senegal, Swaziland, Lesotho, Zambia, Uganda, Burkina Faso and Zimbabwe are considering it.^8 9^ Since most SSA countries are characterised by high levels of informality in the labour market, health insurance mechanisms are predominantly voluntary (either de facto or de jure).

Given the increasing interest by LMICs in SSA to adopt health insurance, in this paper we examine the performance of health insurance schemes in these countries. Specifically, we use data from the Demographic and Health Surveys (DHS) from 36 SSA countries to examine the level and equity of health insurance coverage in health insurance enrolment, as well as factors that contribute to observed inequality in coverage. We use this evidence to address ongoing debates about key design features of UHC schemes. Health insurance schemes are often viewed as a way to raise additional resources for the health sector through member contributions. We explore if this is a reasonable expectation in light of the experience of existing health insurance schemes. Furthermore, the distribution of insurance coverage across wealth quintiles is an important consideration given that equity is one of the central tenets of UHC.^10^ When UHC cannot be achieved immediately, it is critical that progress is made equitably.^11^ In this context, we examine how well health insurance schemes in SSA fare in terms of equity.

## Methods

We analysed secondary datasets from the DHS for 36 SSA countries. The decision to include a country in the analysis was informed by (1) the availability of DHS survey dataset that was collected after the year 1999 and (2) availability of data on health insurance coverage in the country’s latest standard DHS dataset as of 6 October 2020. The DHS is a household survey that uses a two-stage cluster sampling design to collect nationally representative data on marriage, fertility, family planning, reproductive health and child health every 5 years.^12^ In some countries, the DHS collects data on whether respondents are enrolled in any health insurance scheme, as well as in specific health insurance schemes. The DHS methods (survey design and tools) are standardised, making the survey data comparable across countries and time. We also conducted a desk review of the insurance schemes in study countries (where information was available) to obtain information on key characteristics of publicly owned health insurance schemes, specifically (1) funding mechanism (premiums, general revenue allocation and earmarked taxes), and (2) eligible population (formal sector (private or public sector) and informal sector individuals). Table 1 outlines the countries whose data were analysed, and key characteristics relevant to insurance coverage.

**Table 1:** Analysis of countries and relevant characteristics

Country	Poverty rate* (%)	Unemployment rate† (%)	Informal employment rate‡ (%)	Characteristics of the country’s publicly owned insurance scheme	
				Revenue source/contribution mechanism	Population eligible for enrolment
Angola	30.1	7.3	42.1	No publicly owned insurer	
Benin	49.5	1.0	53.4	Member contributions Taxes on tobacco and alcohol Government contributions as an employer	Government employees Private sector formal workers Informal sector workers
Burkina Faso	40.1	3.0	18.0	Member contributions	Formal sector Elderly and people with disability
Burundi	64.6	1.6	10.0	Member contributions General revenue allocation	Government employees Informal sector workers
Cameroon	37.5	4.5	91.0	No publicly owned insurer	
Chad	38.1	1.1	66.3		
Comoros	18.0	20.1	30.0		
Congo	46.5	11.4	–		
Congo Democratic Republic	63.9	3.6	98.6		
Cote d'Ivoire	46.3	9.2	92.8	Member contributions Government contributions as an employer	All population: Government employees Private sector formal workers Informal sector workers The poor
Eswatini	63.0	25.7	17.7	No publicly owned insurer	
Ethiopia	29.6	5.4	46.7	Member contribution Government contributions as an employer	Government employees Private sector formal employees
Gabon	32.7	18.5	38.0	General revenue allocation 10% tax on mobile phones Compulsory levy called the Special Solidarity Contribution Member contributions	Government employees Private sector formal workers Informal sector workers The poor
Gambia	24.2	29.8	76.5	No publicly owned insurer	
Ghana	48.4	5.8	92.1	2.5% VAT Member contribution Government revenue allocation An earmarked portion of social security taxes from formal sector workers	Government employees Private sector formal workers Informal sector workers The poor
Guinea	36.1	4.5	77.6	No publicly owned insurer	
Kenya	36.1	11.0	77.9	Member contributions Government contributions as an employer Government revenue allocation	Government employees Private sector formal workers Informal sector workers The poor
Lesotho	57.1	29.2	34.9	No publicly owned insurer	
Liberia	63.8	4.0	86.7	Member contributions Employer contributions Government contributions as an employer	Government employees Private sector formal employees
Madagascar	70.7	2.4	12.0	No publicly owned insurer	
Malawi	50.7	6.7	89.0	No publicly owned insurer	
Mali	43.6	9.7	42.6	Member contribution Government revenue allocation Government contributions as an employer	Government employees Private sector formal employees The poor
Mozambique	46.1	24.5	95.7	General revenue allocation Donor funding	All population: Government employees Private sector formal workers Informal sector workers The poor
Namibia	28.7	22.3	67.0	Government contributions as an employer Member contributions	Government employees
Niger	46.0	2.6	95.4	Government contributions as an employer Member contributions	Government employees
Nigeria	44.5	5.5	–	Member contributions Employer contributions Government contributions as an employer	All population: Government employees Private sector formal workers Informal sector workers The poor
Rwanda	39.1	2.6	73.4	Member contribution Employer contribution General government revenue allocation Donor funding	All population: Government employees Private sector formal workers Informal sector workers The poor
Sao Tome and Principe	66.2	13.7	73.0	No publicly owned insurer	
Senegal	46.7	9.5	93.4	Member contribution General government revenue allocation	Government employees and retirees
Sierra Leone	52.9	2.8	9.0	Member contribution Employer contribution	Government employees Private sector formal workers
South Africa	18.8	27.6	27.1	Government taxes	
Tanzania	28.2	2.7	90.8	Member contribution Employer contribution	Government employees Private sector formal employees Informal sector workers
Togo	55.1	6.2	84.0	Member contribution Government contributions as an employer Employer contribution	Government employees Private sector formal employees
Uganda	19.5	2.3	91.7	No publicly owned insurer	
Zambia	54.4	7.4	74.4	No publicly owned insurer	
Zimbabwe	72.3	5.3	85.6	Member contribution Government contributions as an employer	Government employees

*Poverty rate refers to the proportion of the population who lives below the international poverty line, that is, US$1.90 per day. This was obtained from the World Bank (https://data.worldbank.org/indicator/SI.POV.DDAY?end=2017&start=2017&view=bar).

†Unemployment rate refers to the proportion of individuals in the labour force who do not have any form of employment. This was obtained from the World Bank (https://data.worldbank.org/indicator/SL.UEM.TOTL.NE.ZS).

‡Informality rate refers to the proportion of employed individuals in a country whose employment is in the informal sector of the economy—this was computed from DHS data.

DHS, Demographic and Health Surveys; VAT, value-added tax.

### Data analysis

#### Level of health insurance

We appended male and female standard DHS datasets into country-specific datasets and then pooled these country-specific datasets into one multicountry dataset. We examined the distribution of the dichotomous variable of health insurance enrolment in relation to other variables suggested in the literature. The country-level analysis incorporated the weight, clustering and stratification variables provided by DHS, and we used the *svyset* command, to account for the study design. For the pooled analysis, we de-normalised the weights for the country-specific dataset prior to pooling into a multicountry dataset by applying country-specific weights calculated by comparative population sizes at the midpoint time of the survey (usually 1 July—using population data from the World Population Prospects; https://population.un.org/wpp/Download/Standard/Population/). We computed the weighted mean of health insurance coverage by country, for any type of health insurance, as well as for specific types of health insurance at the country-level and the mean of health insurance coverage with any type of health insurance for 35 countries (excluding Rwanda and applying the pooled weights).

#### Measuring inequality in health insurance coverage

We used four approaches to assess income-related inequality in health insurance coverage. First, we computed slope index of inequality (SII). The SII is a complex, weighted measure of inequality computed from a regression model where the whole population is ranked from the most disadvantaged (at rank 0) to the most advantaged (at rank 1). The SII was preferred as an absolute measure of inequality compared with other measures such as the range as it takes into consideration not only the two extreme quintiles (the richest—Q5 and the poorest—Q1) but also all the other subgroups (Q2, Q3 and Q4). Second, we calculated the rich–poor ratio, which divides the percentage of health insurance coverage among individuals in Q5 to the percentage coverage among individuals in Q1. While a rich–poor ratio does not consider the distribution of the variable of interest across the entire population, it can easily be interpreted by lay readers and policymakers.^13^ Third, we constructed concentration curves of health insurance coverage. A concentration curve is a plot of the cumulative percentage of a variable of interest (health insurance coverage) (y-axis) against the cumulative proportion of the population, ranked by socioeconomic status, from the poorest to the richest (x-axis).^14^ The concentration curve is a 45° line (line of equality) when every individual, irrespective of their socioeconomic status, receives the same value of the variable of interest.^14^ A concentration curve that lies above (below) the line of equality indicates that the variable of interest is concentrated among the poor (rich).^14^ The further the curve is above (below) the line of equality, the higher the pro-poor (pro-rich) inequality.^14^ Fourth, while the concentration curve signals the presence (or absence) and the direction of inequalities, it does not reveal the magnitude. We, therefore, computed the concentration index (CIX), defined as twice the area between the concentration curve and the line of equality.^14^ A CIX of zero denotes equality, while a negative (positive) CIX indicates a pro-poor (pro-rich) distribution of the health variable.^14^ We used the Wagstaff’s normalised CIX because our variable of interest is a dichotomous variable with a lower bound of 0 and an upper bound of 1.^15^

#### Decomposition of inequality in health insurance coverage

Wagstaff *et al* have demonstrated that the CIX (C) can be decomposed into contributions of individual factors to income-related inequality in a variable of interest, in which each contribution is the product of the elasticity (βk is the coefficient of X from the regression model and X̄_k_ is the mean of *x_k_*) of the variable of interest with respect to that factor, and the degree of income-related inequality in that factor (C_k_) and the last term, GC_ε_ /μ being the residual contribution for the error term (unmeasurable component) ε, as below.^16^

$$C=\;\sum_{K}\left(\beta_{K\;\;}{\overset{-}{X}}_{K\;\;}/\mu \right)C_{K\;\;}+G\beta C_{E\;\;}/\mu$$

The elasticity of a variable is a unit-free measure of association interpreted as the percentage change in the dependent variable (health insurance coverage in this case) associated with a percentage change in the predictor variable.^17^ To examine the factors contributing to observed inequality in health insurance coverage, we used a country-stratified, survey-weighted generalised linear model (GLM) with a binomial logit link to decompose the computed CIX of health insurance coverage. The GLM was preferred due to the binary nature of our outcome variable (whether an individual had or did not have any form of health insurance) and has been widely used to decompose socioeconomic-related inequalities in health.^17–19^ A positive (negative) contribution indicates that a factor increases pro-rich (pro-poor) inequality of health insurance coverage. To assess whether the contributions were statistically significant, we computed 95% CIs from bootstrapped SEs of the absolute contributions. We identified the factors to include in the regression model from existing literature on factors that are associated with individual enrolment to health insurance schemes.^20–22^ Data analyses were performed in STATA V.14, and all estimates were weighted to take into account the complex study design employed in the DHS.

## Results

**Figure 1:**
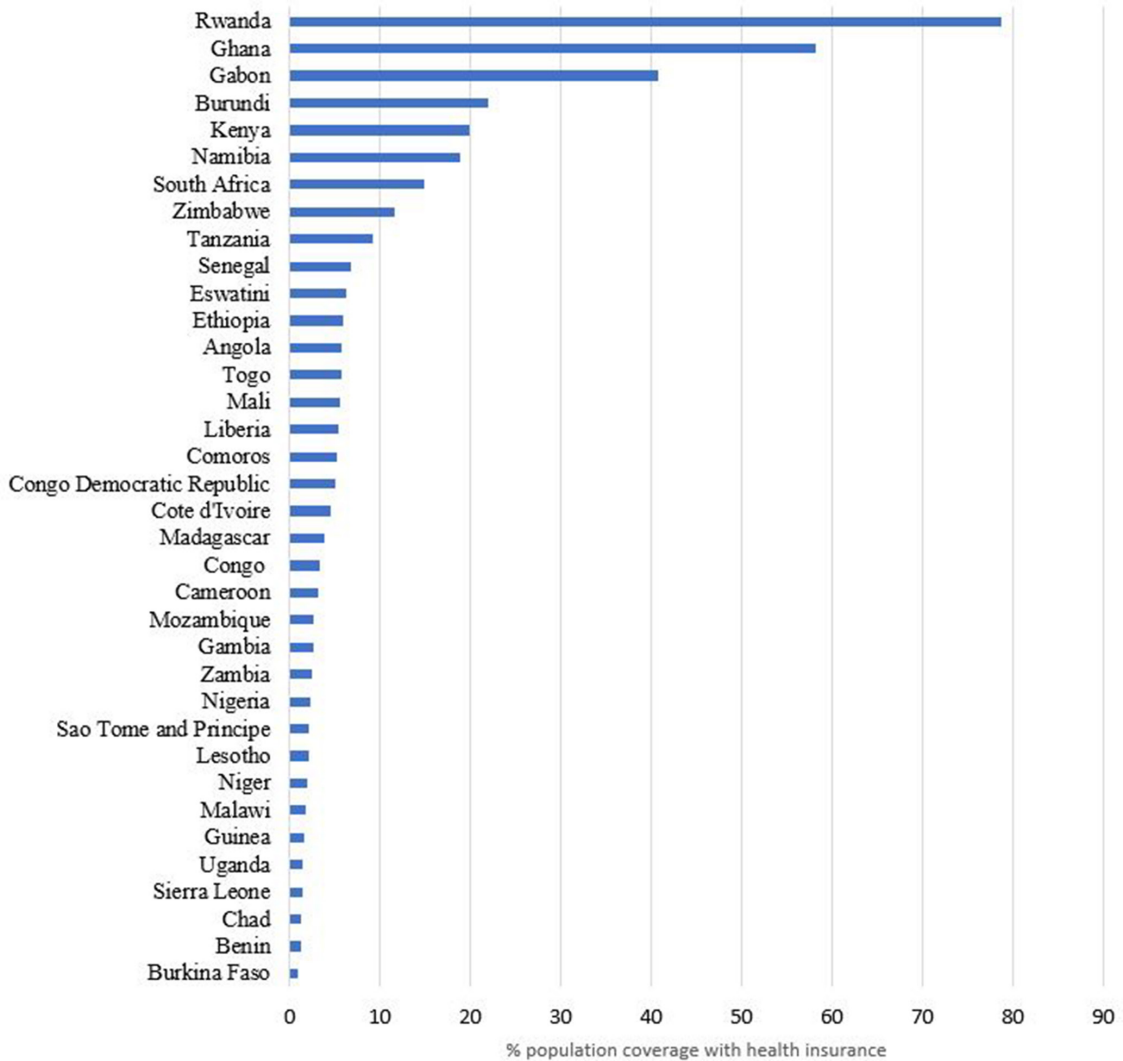
Mean level of health insurance coverage with any form of health insurance in 36 sub-Saharan African countries.

Figure 1 shows the mean level of health insurance coverage with any form of health insurance, while table 2 shows the mean level of health insurance coverage by type of health insurance in the 36 countries examined. The level of coverage with any form of health insurance varied across the countries ranging from 0.9% (95% CI: 0.7% to 1.1%) in Burkina Faso to 78.7% (95% CI: 77.5% to 79.9%) in Rwanda. The weighted mean level of coverage for the pooled dataset of 36 countries was 7.9% (95% CI: 7.8% to 7.9%).

**Table 2:** Mean level of coverage by type of health insurance in 36 SSA countries

Country	Survey year	Total N	% coverage with any insurance (95% CI)	% coverage with national (public) health insurance (95 % CI)	% coverage with private or employer health insurance (95 % CI)	% coverage with community-based health insurance (CBHI) (95 % CI)
Rwanda	2014	12 699	78.7 (77.5 to 79.9)	5.0 (4.5 to 5.6)	0.7 (0.5 to 1.0)	76.4 (75.2 to 77.6)*
Ghana	2014	13 780	58.2 (56.2 to 60.1)	57.7 (56.9 to 58.5)	0.8 (0.7 to 1.0)	0.1 (0.0 to 0.1)
Gabon	2012	14 043	40.8 (38.20 to 43.5)	–	–	–
Burundi	2017	24 821	22.0 (20.7 to 23.2)	–	16.4 (15.9 to 16.9)	5.2 (5.0 to 5.5)
Kenya	2014	27 548	19.9 (18.7 to 21.1)	15.9 (15.5 to 16.3)	4.1 (3.8 to 4.4)	0.3 (0.2 to 0.4)
Namibia	2013	14 492	18.8 (17.1 to 20.6)	5.0 (4.7 to 5.4)	14.0 (13.5 to 14.6)	–
South Africa	2016	7811	14.9 (12.9 to 17.1)	–	–	–
Zimbabwe	2015	18 351	11.6 (10.1 to 13.3)	0.3 (0.2 to 0.3)	10.2 (9.7 to 10.6)	1.0 (0.82 to 1.1)
Tanzania	2015	16 778	9.2 (8.2 to 10.2)	1.6 (1.4 to 1.8)	3.1 (2.9 to 3.4)	4.5 (4.1 to 4.8)
Senegal	2010	20 615	6.9 (5.9 to 8.0)	0.6 (0.5 to 0.7)	2.0 (1.8 to 2.2)	1.6 (1.6 to 1.9)
Eswatini	2006	9131	6.2 (5.4 to 7.2)	–	2.4 (2.1 to 2.7)	–
Ethiopia	2016	28 371	5.9 (4.7 to 7.2)	0.8 (0.7 to 0.9)	0.6 (0.5 to 0.7)	4.5 (4.2 to 4.7)
Angola	2016	20 063	5.8 (5.1 to 6.6)	–	–	–
Togo	2013	13 951	5.8 (4.1 to 6.6)	0.2 (0.1 to 0.3)	1.5 (1.3 to 1.7)	0.4 (0.3 to 0.5)
Mali	2018	15 137	5.6 (4.5 to 6.8)	1.6 (1.2 to 2.2)	1.8 (1.3 to 2.3)	2.6 (2.1 to 3.1)
Liberia	2013	13 340	5.3 (4.1 to 7.0)	1.7 (1.2 to 2.3)	4.6 (4.3 to 5.0)	1.3 (0.8 to 1.9)
Comoros	2012	7485	5.3 (4.4 to 6.4)	0.4 (0.1 to 0.8)	2.6 (2.2 to 2.9)	2.5 (2.0 to 3.0)
Congo Democratic Republic	2013	27 465	5.0 (4.2 to 5.9)	0.04 (0.02 to 0.1)	3.7 (3.5 to 3.9)	1.2 (1.1 to 1.3)
Cote d'Ivoire	2011	15 165	4.6 (3.6 to 5.8)	1.7 (1.1 to 2.3)	2.2 (1.9 to 2.4)	3.6 (3.0 to 4.3)
Madagascar	2008	17 085	3.8 (3.2 to 4.6)	1.2 (0.7 to 1.7)	2.9 (2.6 to 3.1)	1.9 (1.4 to 2.4)
Congo	2011	15 955	3.3 (2.8 to 3.9)	0.3 (0.2 to 0.5)	2.1 (1.9 to 2.3)	1.1 (0.9 to 1.3)
Cameroon	2018	20 505	3.2 (2.8 to 3.7)	0.6 (0.5 to 0.8)	2.1 (1.9 to 2.3)	0.5 (0.4 to 0.8)
Mozambique	2011	17 780	2.7 (2.4 to 3.1)	–	–	–
Gambia	2013	14 030	2.62 (2.2 to 3.2)	–	2.5 (2.2 to 2.7)	–
Zambia	2018	25 815	2.54 (2.06 to 3.13)	0.03 (0.01 to 0.06)	2.13 (1.95 to 2.31)	0.2 (0.2 to 0.3)
Nigeria	2018	56 155	2.3 (2.0 to 2.7)	0.6 (0.5 to 0.8)	1.9 (1.8 to 2.0)	0.86 (0.7 to 1.1)
Sao Tome and Principe	2008	4898	2.2 (1.7 to 2.7)	0.7 (0.2 to 1.2)	1.1 (0.8 to 1.4)	0.9 (0.4 to 1.5)
Lesotho	2014	9552	2.09 (1.70 to 2.56)	–	1.4 (1.2 to 1.7)	0.4 (0.2 to 0.5)
Niger	2012	15 074	2.03 (1.68 to 2.5)	2.1 (1.4 to 2.7)	1.1 (0.9 to 1.2)	2.2 (1.6 to 2.9)
Malawi	2016	32 040	1.8 (1.3 to 2.6)	–	1.8 (1.7 to 1.9)	–
Guinea	2018	14 991	1.6 (1.21 to 2.2)	0.2 (0.1 to 0.2)	1.3 (1.2 to 1.5)	0.1 (0.1 to 0.2)
Uganda	2016	23 842	1.5 (1.3 to 1.8)	0.0 (0.0 to 0.1)	1.1 (0.9 to 1.2)	0.4 (0.3 to 0.5)
Sierra Leone	2013	23 887	1.5 (1.1 to 1.9)	0.5 (0.3 to 0.7)	0.9 (0.7 to 1.0)	0.6 (0.4 to 0.8)
Chad	2015	11 380	1.2 (0.9 to 1.6)	0.1 (0.0 to 0.2)	0.7 (0.6 to 0.9)	0.4 (0.2 to 0.7)
Benin	2018	23 523	1.2 (0.1 to 1.4)	0.1 (0.1 to 0.2)	0.8 (0.7 to 0.9)	0.2 (0.2 to 0.3)
Burkina Faso	2010	24 382	0.9 (0.7 to 1.1)	0.6 (0.3 to 0.9)	0.4 (0.3 to 0.4)	0.9 (0.5 to 1.0)
Total†		637 752	7.9 (7.8 to 7.9)	–	–	–

– means data are not available.

*Rwanda’s CBHI is now managed by the national government rather than local communities and is more suitably classified as a national public health insurance scheme rather than a CBHI.

†Pooled data.

SSA, sub-Saharan Africa.

Table 3 shows the distribution of coverage with any form of health insurance by selected sociodemographic variables status. Across all countries, health insurance coverage increased with employment status, exposure to media, level of education and socioeconomic status.

**Table 3:** Level of coverage with any type of health insurance across selected sociodemographic factors in 36 SSA countries

Country	Employment status			Residence		Exposure to media			Level of education				Socioeconomic status				
	Unemployed	Informal	Formal	Rural	Urban	Not at all	Less than once a week	At least once a week	No education	Primary	Secondary	Higher	Poorest	Poorer	Middle	Richer	Richest
Angola	6.0	4.2	8.4	2.3	7.3	3.6	4.8	7.1	2.9	3.0	7.1	22.1	1.2	3.0	3.4	6.2	12.3
Benin	1.1	0.7	2.2	0.6	2.0	0.2	0.5	2.1	0.2	0.9	2.0	10.3	0.1	0.4	0.4	0.7	3.8
Burkina Faso	0.9	0.5	2.0	0.2	2.5	0.0	0.3	1.4	0.2	0.6	2.8	18.6	0.1	0.2	0.2	0.2	2.9
Burundi	18.1	20.4	49.6	21.5	25.1	15.9	21.9	28.7	19.8	21.3	23.7	56.7	11.2	16.8	21.0	26.0	32.6
Cameroon	2.4	3.2	4.4	0.9	5.1	0.5	2.4	5.2	0.3	1.0	3.4	14.5	0.0	0.7	1.1	2.9	9.2
Chad	1.0	0.8	2.2	0.3	3.7	0.1	0.8	3.8	0.1	0.9	2.5	16.7	0.0	0.3	0.7	0.2	4.0
Comoros	4.3	3.8	12.3	3.5	8.7	1.6	4.0	6.5	1.5	3.9	5.6	17.0	1.3	1.6	4.4	5.8	11.8
Congo	3.1	2.8	4.3	1.3	4.2	2.0	3.2	4.4	0.6	0.8	3.5	10.6	0.9	1.1	2.1	3.4	8.1
Congo Democratic Republic	6.8	2.1	8.8	1.7	10.4	1.7	3.9	9.0	1.5	1.3	6.3	22.7	0.9	0.7	1.7	4.1	14.9
Cote d'Ivoire	6.2	2.4	6.6	1.4	7.6	0.3	1.4	7.0	0.9	2.3	10.9	27.3	0.2	1.2	1.3	3.8	13.4
Eswatini	3.0	5.8	17.1	2.9	15.1	7.6	4.5	5.5	3.4	2.6	5.9	26.5	1.3	0.9	2.3	4.2	17.4
Ethiopia	3.1	7.5	5.5	6.0	5.4	4.8	7.1	6.5	6.5	4.9	5.7	8.0	2.8	4.7	8.0	7.9	5.4
Gabon	40.9	36.2	46.8	55.2	38.9	39.2	39.4	42.9	14.4	42.7	39.1	58.7	56.3	34.6	32.3	35.6	49.6
Gambia	1.8	1.8	5.5	0.7	4.0	0.6	1.1	3.2	0.7	1.0	3.8	12.1	0.2	0.8	1.0	2.6	6.7
Ghana	63.0	52.6	62.0	56.3	59.9	54.1	56.5	58.9	58.3	50.9	58.0	73.6	59.0	53.1	53.8	58.3	65.1
Guinea	1.5	1.0	3.8	0.4	3.6	0.1	1.1	2.7	0.4	1.0	3.7	9.2	0.1	0.1	0.5	1.2	5.3
Kenya	9.6	18.1	52.8	13.9	28.0	3.0	9.0	22.6	2.6	10.8	22.7	55.9	3.2	8.0	13.9	23.4	39.5
Lesotho	7.8	1.4	5.1	0.9	4.2	0.1	0.4	3.0	0.0	0.8	1.6	11.5	0.0	0.4	0.6	1.4	6.0
Liberia	5.0	4.4	7.4	4.2	6.1	1.9	3.5	7.7	2.2	4.3	6.1	20.4	0.7	1.3	5.4	7.0	10.1
Madagascar	4.6	2.7	24.5	2.1	12.2	2.5	4.0	7.1	0.3	1.3	7.9	26.0	0.3	0.3	0.7	2.2	12.9
Malawi	1.2	0.8	9.7	0.7	7.1	0.2	1.2	3.6	0.4	0.4	2.8	26.1	0.1	0.1	0.4	0.5	6.7
Mali	5.2	2.3	11.3	2.9	13.2	1.8	2.1	7.7	1.5	3.1	13.3	45.5	1.7	0.9	0.7	4.2	17.7
Mozambique	2.0	2.1	6.5	1.6	4.6	1.7	2.1	3.3	1.4	1.8	5.3	23.6	0.9	0.9	1.5	2.6	6.6
Namibia	8.8	22.9	33.4	8.4	26.9	2.5	10.0	22.9	4.8	5.0	18.1	61.9	1.6	4.2	10.4	19.8	47.8
Niger	1.6	0.9	4.1	0.6	7.5	0.4	0.7	4.0	0.6	2.1	9.9	26.2	0.2	0.2	0.3	0.7	7.3
Nigeria	1.5	1.8	3.4	1.0	4.0	0.3	1.4	3.5	0.2	0.7	2.1	11.9	0.0	0.2	0.9	2.0	7.0
Rwanda	–	–	–	–	–	–	–	–	–	–	–	–	63.5	75.0	82.4	87.8	88.9
Sao Tome and Principe	1.4	2.4	2.5	2.0	2.3	2.4	2.1	2.0	0.6	1.3	3.5	6.8	0.8	1.2	1.5	1.8	5.0
Senegal	6.4	4.6	11.4	2.3	11.3	1.5	1.6	8.0	1.6	6.6	16.7	39.9	1.3	2.1	4.6	6.9	16.0
Sierra Leone	1.3	0.9	9.2	0.5	3.1	0.4	0.7	2.5	0.4	1.4	2.0	11.6	0.3	0.4	0.7	1.4	3.9
South Africa	8.5	14.8	48.5	8.1	18.1	3.1	8.0	16.8	4.4	4.2	12.4	45.1	2.3	4.0	8.7	16.1	46.4
Tanzania	9.3	7.2	45.1	8.5	10.4	3.6	7.8	11.1	4.8	6.6	15.6	51.2	4.1	5.6	7.3	10.4	14.8
Togo	6.1	5.3	6.4	3.2	8.9	3.1	4.9	8.3	1.2	2.7	8.8	24.8	1.6	1.4	3.8	5.3	13.2
Uganda	0.9	0.9	4.6	1.0	3.0	0.2	0.9	2.0	0.7	0.6	1.6	7.9	0.2	0.5	0.8	1.2	3.9
Zambia	1.0	1.6	8.3	0.6	4.9	1.5	2.5	4.1	1.0	0.2	1.8	23.3	0.0	0.0	0.2	1.3	9.2
Zimbabwe	7.3	8.6	25.9	4.1	24.1	1.4	4.6	17.3	1.8	0.7	9.3	59.9	0.3	0.6	2.0	11.6	33.7

– means data are not available.

SSA, sub-Saharan Africa.

### Inequalities in health insurance coverage

**Figure 2:**
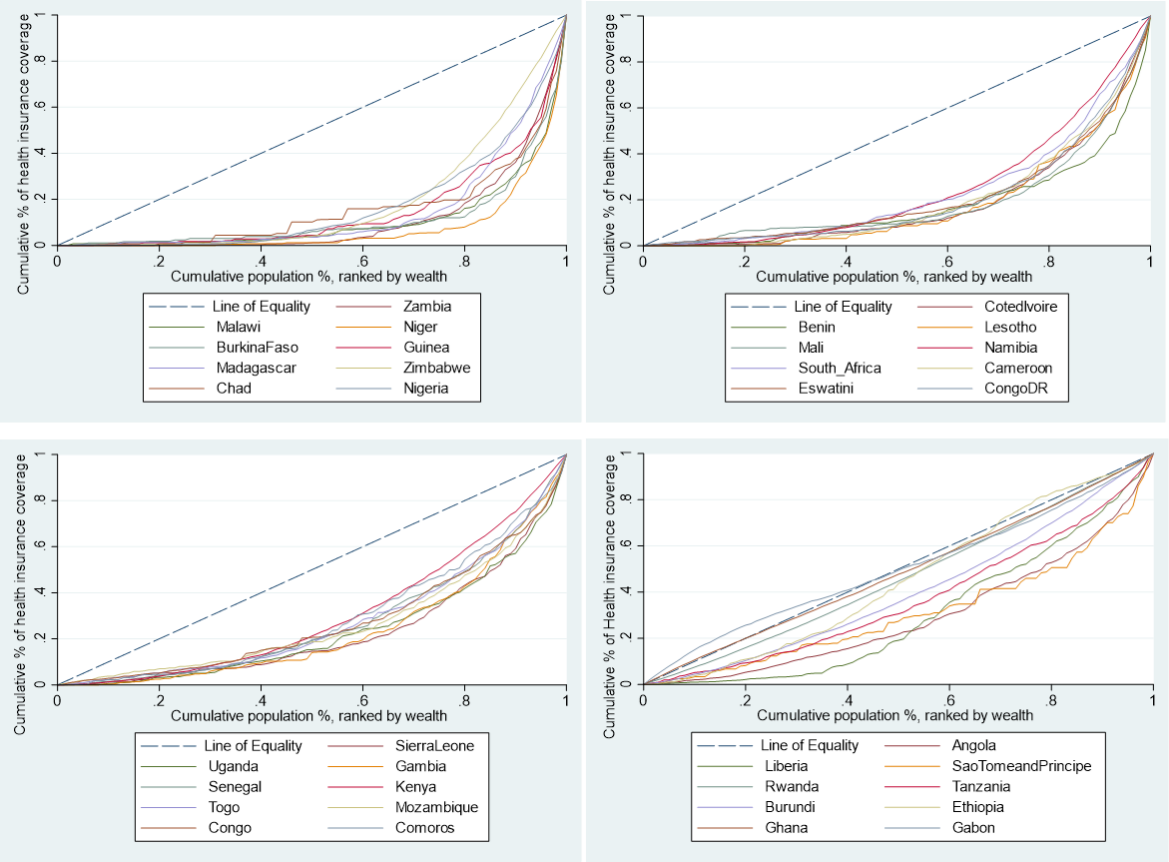
Concentration curves for health insurance coverage, by country, in 36 sub-Saharan African countries (ranked in decreasing inequality).

Figure 2 presents concentration curves for coverage with any form of health insurance for the 36 study countries, while table 4 presents the SII, rich–poor ratios and CIX. Overall, health insurance coverage in the 36 SSA countries was characterised by pro-rich inequality. Health insurance coverage for each type of health insurance was also characterised by pro-rich inequality. There was great variability in the extent of inequality of health insurance coverage across the 36 countries. In absolute terms, Namibia had the largest gap between the richest and the poorest (46.25%); whereas in Gabon, the poorest had a 6.72% higher coverage than the richest. The highest inequalities in coverage with any type of health insurance were observed in Zambia (SII=0.223, CIX=0.80 (95% CI: 0.75 to 0.84; p<0.001) and Malawi (SII=0.11, Q5/Q1=56.08, CIX=0.80 (95% CI 0.53 to 1.07; p<0.001)), while the lowest (or no) inequalities were observed in Gabon (SII=−0.21, Q5/Q1=0.88, CIX=0.00 (95% CI −0.04 to 0.04; p=0.893)). Furthermore, out of the 27 countries that had data on both national/public health insurance and private or employer-provided health insurance, 59% (16) had a lower CIX for the national or public insurance schemes than that of private or employer-provided insurance schemes. This indicates that national/public health insurance schemes can potentially reduce inequalities in health insurance coverage.

**Table 4:** Slope index of inequality (SII), high-to-low (richest/poorest—Q5/Q1) ratios and concentration indices (CIX) of health insurance coverage in 36 SSA countries ranked by CIX—any type of health insurance

	Any type of health insurance			National/public health insurance			Private or employer-provided health insurance			Community-based health insurance		
	SII	Q5/Q1	CIX (95% CI)	SII	Q5/Q1	CIX (95% CI)	SII	Q5/Q1	CIX (95% CI)	SII	Q5/Q1	CIX (95% CI)
Zambia	0.223*** (0.195 to 0.250)	–	0.80*** (0.75 to 0.84)	0.002* (0.000 to 0.004)	–	0.63** (0.25 to 1.00)	0.193*** (0.066 to 0.219)	–	0.80*** (0.75 to 0.84	–	–	0.85*** (0.69 to 1.00)
Malawi	0.105*** (0.089 to 0.121)	56.08	0.80*** (0.53 to 1.07)	–	–	–	0.105*** (0.088 to 0.121)	110.67	0.82*** (0.54 to 1.08)	–	–	–
Niger	0.402*** (0.327 to 0.478)	38.47	0.79*** (0.63 to 0.95)	0.039*** (0.016 to 0.061)	–	0.75*** (0.48 to 1.02)	–	–	0.92*** (0.67 to 1.17)	0.065*** (0.033 to 0.097)	–	0.78*** (0.51 to 1.05)
Burkina Faso	0.052*** (0.038 to 0.067)	36.38	0.73*** (0.55 to 0.91)	0.015** (0.004 to 0.026)	–	0.79 (0.40 to 1.18)	–	–	0.86*** (0.57 to 1.15)	0.016*** (0.010 to 0.023)	13.88	0.70*** (0.45 to 0.95)
Guinea	0.102*** (0.081 to 0.123)	47.73	0.72*** (0.47 to 0.97)	–	–	0.82** (0.36 to 1.29)	0.085*** (0.066 to 0.105)	59.85	0.71*** (0.42 to 0.98)	0.007* (0.001 to 0.012)	13.31	0.75** (0.20 to 1.30)
Madagascar	0.305*** (0.271 to 0.338)	42.90	0.72*** (0.60 to 0.84)	0.014* (0.004 to 0.023)	–	0.74*** (0.32 to 1.15)	0.240*** (0.006 to 0.375)	36.85	0.73*** (0.59 to 0.87)	0.072*** (0.053 to 0.092)	–	0.73*** (0.48 to 0.98)
Zimbabwe	0.540*** (0.519 to 0.560)	140.54	0.71*** (0.69 to 0.73)	0.020*** (0.012 to 0.028)	–	0.60*** (0.44 to 0.76)	0.477*** (0.056 to 0.598)	174.29	0.70*** (0.68 to 0.72)	0.070*** (0.056 to 0.085)	34.50	0.61*** (0.53 to 0.69
Chad	0.062*** (0.043 to 0.080)	–	0.71*** (0.52 to 0.90)	–	–	0.97* (0.10 to 1.84)	0.045*** (0.027 to 0.062)	–	0.75 (0.50 to 1.00)	0.011*** (0.004 to 0.018)	–	0.52 (0.22 to 0.82)
Nigeria	0.135*** (0.125 to 0.145)	348.00	0.67*** (0.57 to 0.77)	0.002** (0.001 to 0.003)	–	0.54 (−0.17 to 1.25)	0.124*** (0.014 to 0.134)	–	0.70*** (0.58 to 0.82)	0.013*** (0.010 to 0.016)	–	0.56*** (0.31 to 0.81)
Cote d'Ivoire	0.169*** (0.148 to 0.190)	83.88	0.66*** (0.50 to 0.82)	0.019*** (0.008 to 0.031)	–	0.74** (0.31 to 1.17)	0.062*** (0.049 to 0.174)	45.50	0.55*** (0.43 to 0.67)	0.118*** (0.096 to 0.140)	–	0.75*** (0.48 to 1.02)
Benin	0.071*** (0.058 to 0.085)	54.57	0.65*** (0.52 to 0.80)	0.010** (0.004 to 0.016)	–	0.79 (0.58 to 0.99)	0.075*** (0.056 to 0.094)	74.00	0.75*** (0.58 to 0.93)	0.005*** (0.003 to 0.008)	13.33	0.31** (0.08 to 0.53)
Lesotho	0.094*** (0.073 to 0.116)	–	0.66*** (0.50 to 0.82)	–	–	–	0.071*** (0.051 to 0.091)	–	0.67*** (0.51 to 0.83)	0.019** (0.006 to 0.033)	–	0.72*** (0.35 to 1.09)
Mali	0.265*** (0.238 to 0.292)	10.44	0.65*** (0.51 to 0.79)	0.014** (0.004 to 0.023)	111.80	0.75*** (0.52 to 0.99)	0.085*** (0.066 to 0.204)	–	0.59*** (0.39 to 0.79)	0.141*** (0.117 to 0.165)	9.31	0.63*** (0.48 to 0.78)
Namibia	0.574*** (0.553 to 0.595)	30.46	0.63*** (0.57 to 0.69)	0.116*** (0.101 to 0.131)	17.14	0.35*** (0.27 to 0.43)	0.499*** (0.076 to 0.522)	36.76	0.64*** (0.58 to 0.70)	–	–	–
South Africa	0.441*** (0.409 to 0.473)	19.99	0.63*** (0.60 to 0.66)	–	–	–	–	–	–	–	–	–
Cameroon	0.136*** (0.120 to 0.152)	–	0.63*** (0.54 to 0.71)	0.033*** (0.024 to 0.043)	–	0.65*** (0.48 to 0.82)	0.089*** (0.076 to 0.102)	–	0.64*** (0.53 to 0.74)	0.024*** (0.016 to 0.032)	–	0.60*** (0.37 to 0.83)
Eswatini	0.316*** (0.283 to 0.349)	13.30	0.63*** (0.59 to 0.67)	–	–	–	0.127*** (0.004 to 0.351)	11.04	0.50*** (0.42 to 0.58)	–	–	–
Congo Democratic Republic	0.193*** (0.175 to 0.210)	16.33	0.63*** (0.53 to 0.73)	0.002* (0.000 to 0.004)	–	0.75** (0.20 to 1.30)	0.199*** (0.078 to 0.120)	31.25	0.71*** (0.59 to 0.83)	0.019*** (0.013 to 0.024)	4.64	0.34*** (0.18 to 0.50)
Sierra Leone	0.061*** (0.050 to 0.071)	17.41	0.55*** (0.49 to 0.61)	0.016*** (0.011 to 0.021)	–	0.60*** (0.46 to 0.74)	0.041*** (0.033 to 0.050)	24.20	0.60*** (0.52 to 0.68)	0.008*** (0.004 to 0.012)	5.36	0.33*** (0.21 to 0.45)
Uganda	0.052*** (0.043 to 0.062)	20.26	0.54*** (0.48 to 0.60)	0.003 (0.000 to 0.006)	–	0.61*** (0.16 to 1.06)	0.055*** (0.043 to 0.068)	30.64	0.70*** (0.62 to 0.78)	0.005*** (0.002 to 0.008)	5.43	0.12* (0.00 to 0.24)
Gambia	0.109*** (0.090 to 0.127)	28.96	0.54*** (0.42 to 0.67)	–	–	–	0.111*** (0.092 to 0.130)	45.79	0.56*** (0.44 to 0.68)	–	–	–
Senegal	0.171*** (0.156 to 0.187)	12.71	0.49*** (0.45 to 0.53)	0.018*** (0.012 to 0.024)	40.25	0.55*** (0.45 to 0.65)	0.053*** (0.043 to 0.162)	21.83	0.55*** (0.49 to 0.61)	0.052*** (0.043 to 0.061)	12.55	0.43*** (0.37 to 0.49)
Kenya	0.448*** (0.433 to 0.464)	12.44	0.49*** (0.45 to 0.53)	0.362*** (0.346 to 0.377)	12.34	0.44*** (0.40 to 0.48)	0.127*** (0.013 to 0.440)	24.38	0.59*** (0.49 to 0.69)	0.007*** (0.004 to 0.010)	4.09	0.30** (0.10 to 0.50)
Togo	0.150*** (0.132 to 0.169)	8.24	0.48*** (0.44 to 0.52)	0.009** (0.004 to 0.015)	–	0.69*** (0.45 to 0.93)	0.080*** (0.059 to 0.101)	52.00	0.72*** (0.64 to 0.80)	(−0.011*** (-0.016 to −0.005)	0.33	−0.25*** (−0.39 to −0.11)
Mozambique	0.090*** (0.076 to 0.104)	6.99	0.48*** (0.38 to 0.58)	–	–	–	–	–	–	–	–	–
Congo	0.070*** (0.057 to 0.082)	8.66	0.47*** (0.35 to 0.59)	0.009** (0.003 to 0.015)	26.67	0.62** (0.23 to 1.01)	0.065*** (0.052 to 0.078)	49.08	0.58*** (0.44 to 0.72)	0.010** (0.003 to 0.016)	2.17	0.20* (0.00 to 0.40)
Comoros	0.146*** (0.123 to 0.170)	8.81	0.45*** (0.33 to 0.57)	0.008* (0.002 to 0.015)	–	0.56* (0.01 to 1.11)	0.076*** (0.058 to 0.194)	6.89	0.42*** (0.28 to 0.56)	0.057*** (0.042 to 0.073)	9.33	0.43*** (0.25 to 0.61)
Angola	0.103*** (0.090 to 0.116)	10.15	0.44*** (0.35 to 0.53)	–	–	–	–	–	–	–	–	–
Liberia	0.176*** (0.156 to 0.195)	14.68	0.42*** (0.29 to 0.55)	0.010*** (0.005 to 0.015)	23.00	0.39 (−0.002 to 0.78)	0.164*** (0.045 to 0.183)	17.41	0.40*** (0.26 to 0.54)	0.010 (−0.002 to 0.023)	–	0.72* (0.07 to 1.37)
Sao Tome and Principe	0.046*** (0.026 to 0.066)	6.51	0.39*** (0.27 to 0.51)	0.006 (−0.002 to 0.014)	3.67	0.38** (0.09 to 0.67)	0.038*** (0.019 to 0.058)	7.10	0.51*** (0.35 to 0.67)	0.003 (−0.005 to 0.010)	6.55	0.20 (−0.02 to 0.42)
Rwanda	0.329*** (0.304 to 0.353)	1.40	0.34*** (0.03 to 0.38)	0.366*** (0.335 to 0.396)	470.50	0.73*** (0.65 to 0.81)	–	–	0.89*** (0.62 to 1.16)	0.237*** (0.211 to 0.264)	1.29	0.24*** (0.20 to 0.28)
Tanzania	0.130*** (0.114 to 0.146)	3.65	0.29*** (0.25 to 0.33)	0.016*** (0.009 to 0.022)	4.02	0.17*** (0.09 to 0.25)	0.138*** (0.019 to 0.157)	52.81	0.66*** (0.60 to 0.72)	0.013** (0.003 to 0.024)	1.25	0.04* (0.01 to 0.08)
Burundi	0.301*** (0.283 to 0.319)	2.91	0.26*** (0.22 to 0.30)	–	–	–	0.031*** (0.016 to 0.046)	1.23	0.05** (−0.34 to 0.44)	0.421*** (0.396 to 0.446)	99.16	0.74*** (0.64 to 0.84)
Ethiopia	0.045*** (0.037 to 0.053)	1.93	0.10** (0.04 to 0.16)	0.004** (0.001 to 0.007)	1.85	0.11 (−0.03 to 0.25)	0.088*** (0.066 to 0.009)	49.75	0.76*** (0.58 to 0.94)	−0.004 (0.009 to 0.002)	1.09	−0.003 (−0.08 to 0.08)
Ghana	0.075*** (0.047 to 0.103)	1.10	0.07** (0.03 to 0.11)	0.061*** (0.033 to 0.090)	1.08	0.06* (0.02 to 0.10)	0.050*** (0.035 to 0.065)	–	0.72*** (0.37 to 1.07)	0.001 (−0.001 to 0.003)	–	0.62 (−0.20 to 1.44)
Gabon	−0.214*** (−0.243 to −0.185)	0.88	0.00 (−0.04 to 0.04)	–	–	–	–	–	–		–	–
**Overall**	0.11*** (0.11 to 0.11)	4.44	0.38*** (0.35 to 0.)	–	–	–	–	–	–	–	–	–

***P<0.001; **p<0.01; *p<0.05.

– means data are unavailable or zero value among poorest; Q1 poorest quintile; Q5 richest quintile.

SSA, sub-Saharan Africa.

### Decomposition of the CIX

Table 5 shows the decomposition analysis results of inequality with any type of health insurance. These findings reveal that exposure to media, socioeconomic rank and the level of education had the greatest contribution to inequality in coverage with any type of health insurance. Specifically, exposure to media (58.49%), belonging to the richest quintile (41.19%), having secondary education (29.40%) and having higher education (17.74%) significantly increased pro-rich inequalities in health insurance coverage. Age (40 years and above), employment status (informal employment), marital status and primary education cumulatively reduced the pro-rich inequalities in health insurance coverage by 15.61%.

**Table 5:** Contribution of independent factors to the concentration index (CIX) for health insurance coverage in the SSA region

	Elasticity	Individual CIX	Contribution to overall CIX	% contribution to overall CIX
Age category 15–24 (reference)				
Age category 25–39	0.067	0.017*** (0.011 to 0.023)	0.001 (0.001 to 0.002)	0.32*** (0.21 to 0.43)
Age category 40–64	0.099	−0.050*** (−0.057 to −0.043)	0.005 (−0.006 to −0.004)	−1.40*** (−1.58 to −1.22)
Subtotal age			−**0.004**	−**1.08**
Not employed (reference)				
Informally employed	0.127	−0.241*** (−0.253 to −0.229)	−0.031 (−0.039 to −0.024)	−8.21*** (−9.99 to −6.43)
Formally employed	0.059	0.294*** (0.280 to 0.308)	0.020 (0.016 to 0.023)	5.33*** (4.38 to 6.29)
Subtotal employment status			−**0.011**	−**2.88**
Gender (1=male)	−0.029	0.016*** (0.010 to 0.022)	0.000 (−0.001 to 0.000)	−0.13*** (−0.18 to −0.08)
Residence (1=urban)	−0.012	0.778*** (0.761 to 0.794)	−0.010 (−0.042 to 0.028)	−2.56 (−9.67 to 4.54)
Marital status (1=married)	0.073	−0.176*** (−0.184 to −0.168)	−0.014 (−0.017 to −0.009)	−3.61*** (−4.63 to −2.60)
Exposure to media (1=exposed)	0.455	0.452*** (0.438 to 0.465)	0.221 (0.193 to 0.257)	58.49*** (51.43 to 65.54)
Household size >5 (reference)				
Household size 4–5	0.062	0.024*** (0.014 to 0.033)	0.002 (0.001 to 0.003)	0.43*** (0.23 to 0.62)
Household size 1–3	0.056	0.080*** (0.069 to 0.092)	0.005 (0.004 to 0.006)	1.40*** (1.10 to 1.71)
Subtotal household size			**0.007**	**1.83**
No education (reference)				
Primary education	0.047	−0.181*** (-0.195 to −0.167)	−0.009 (-0.014 to −0.003)	−2.39*** (−3.95 to −0.84)
Secondary education	0.264	0.384*** (0.371 to 0.396)	0.111 (0.098 to 0.125)	29.40*** (25.41 to 33.39)
Higher education	0.082	0.665*** (0.637 to 0.693)	0.067 (0.061 to 0.073)	17.74*** (16.03 to 19.45)
Subtotal education			**0.169**	**44.75**
Poorest (reference)				
Poorer	−0.003	−0.574*** (−0.591 to −0.556)	0.002 (−0.010 to 0.018)	0.48 (−3.73 to 4.69)
Middle	0.034	−0.101*** (−0.118 to −0.084)	−0.004 (−0.006 to −0.001)	−0.97* (−1.61 to −0.33)
Rich	0.074	0.367*** (0.349 to 0.384)	0.029 (0.015 to 0.040)	7.59*** (4.54 to 10.65)
Richest	0.156	0.947*** (0.929 to 0.966)	0.156 (0.118 to 0.185)	41.19*** (33.86 to 48.51)
Subtotal socioeconomic			**0.183**	**48.29**
Residual			−**0.163** **(−0.205 to −0.126**)	−43.09*** (−55.62 to −30.56)
Overall CIX			0.38*** (0.37 to 0.38)	**100%**

***P<0.001; **p<0.01; *p<0.05.

SSA, sub-Saharan Africa.

## Discussion

As LMICs reform their health systems to achieve UHC, evidence on the performance of alternative health financing mechanisms is critical. This study analysed population coverage with health insurance as a financing scheme in 36 SSA countries. All these countries have other financing schemes that also provide population coverage, and hence the results presented here offer only a partial view of population coverage with health financing systems in these countries. From the results presented in this paper, it is evident that the level of health insurance coverage across SSA countries is very low. Only 8 of the 36 countries examined had a mean level of insurance coverage with any type of health insurance of above 10%, while only 4 had a coverage level of above 20%. This low level of coverage persists regardless of the type of health insurance (public, private and CBHI). The low coverage of health insurance in the study countries is perhaps explained by the fact that these countries are characterised by high levels of informal labour markets and a high incidence of poverty (table 1). International evidence has shown that it is problematic to enrol, retain and collect insurance premiums from individuals in the informal sector.^6 8^ While making enrolment mandatory is theoretically an option, it is practically difficult to enforce. Most SSA countries have hence taken a voluntary approach to covering individuals in the informal sector with health insurance; these individuals are expected—and encouraged—to enrol for the scheme and make a contribution.^23 24^ Even in instances where the schemes are de jure mandatory, such as in Kenya, the inability to enforce makes these schemes de facto voluntary.^25^ ‘Bottom-up’ CBHI schemes were seen as a way to expand insurance coverage to the informal sector, and have been implemented for this reason in many SSA countries, with considerable external support.^8 26^ However, they have yielded mixed results in terms of boosting coverage. As a health financing mechanism, therefore, health insurance that is de facto voluntary for the majority of the population is clearly not effective in achieving population coverage at scale and mobilising sufficient revenues in settings with high poverty and informal labour markets. LMICs outside of Africa that have achieved relatively high levels of health insurance coverage have one characteristic in common; their public health insurance schemes are significantly funded by general revenues rather than premium contributions. For example, Asian countries with high health insurance coverage such as Vietnam, the Philippines and Thailand all have social health insurance schemes that are characterised by significant tax funding.^21^

Our findings also show that health insurance in SSA countries is highly inequitable. This inequality is not only seen in private forms of health insurance but also in publicly owned health insurance schemes; however, public health insurance schemes were characterised by lower inequality than private schemes. Decomposition of the CIX reveals that exposure to media was the greatest contributor to the pro-rich inequality in health insurance coverage. Exposure to media is a proxy for individual socioeconomic status given that richer individuals and households have better access to media. Furthermore, the media is often used to pass health insurance information and mobilise people into joining health insurance schemes. Individual social economic status was the second highest contributor to pro-rich inequality in health insurance coverage. This is consistent with literature that shows that enrolment with health insurance is positively correlated with the ability to pay (socioeconomic status). This highlights the implication of the choice of contribution mechanisms of health financing schemes. Schemes that require individuals to make contributions in order to access benefits select for higher socioeconomic individuals and hence promoting inequality. This perhaps explains why the African countries with the least inequality in health insurance coverage (Gabon, Ghana, Rwanda) also happen to be the ones whose public health insurance schemes have significant funding from general tax revenues, rather than individual member premium contributions. The third highest contributor to pro-rich inequality in health insurance coverage was the level of education. This can be explained by the fact that better-educated individuals are more likely to enrol to a health insurance scheme, and also that high levels of education are concentrated among the rich. These findings highlight the inequality of these factors (education and incomes) in the study countries and the fact that they, in turn, drive inequality in health insurance coverage.

The inequality in health insurance coverage in SSA is also likely driven by the pattern of the introduction of publicly owned health insurance schemes. These schemes have typically been characterised by a phased introduction that begins with government officials, then formal workers, and finally those in the informal sector, and or the poor.^8^ Where the introduction of interventions has started with the well-off in society, it has resulted in entrenching inequalities in coverage.^27^ Further, when efforts to specifically target the poor with social health insurance are fragmented, contributory schemes have been shown to remain inequitable.^28^

### Policy implications

From the foregoing, it is evident that SSA countries will not achieve UHC using health insurance that is de facto voluntary as a health financing scheme. This is because of the challenges presented by high levels of poverty and informality of labour markets. The combination of these contextual realities reduces the proportion of the population that has the ability to pay insurance premiums and also reduces the capacity of insurance agencies to enforce mandatory premium contributions among those with the ability to pay.^8^ As a result, these schemes are characterised by lower population coverage, low retention and adverse selection which compromises their equity, efficiency and financial sustainability.^8^ SSA countries can learn not only from countries outside SSA but also from the few SSA countries that have rapidly increased health insurance coverage and are characterised by less inequities in health insurance coverage. Three out of the four countries (Rwanda, Ghana and Gabon) that have health insurance coverage levels greater than 20% all have one thing in common; they have publicly owned health insurance systems that are significantly tax funded, as opposed to dependence on voluntary contributions (table 1). The CBHI programme in Rwanda is in practice centrally managed by a national government agency, is mandatory for all members of the informal sector and covers over three-quarters of the country’s population. Under the scheme, all poor people receive full government subsidies while some informal sector individuals receive partial subsidies. The scheme is significantly financed by non-contributory mechanisms (donor funding and general taxes).^8 29^ Ghana’s National Health Insurance scheme is predominantly financed by a combination of government allocation from general tax revenues and an earmarked tax (2.5% of value-added tax and 2.5% of social security contributions).^30^ Gabon social health insurance scheme has mixed sources of financing, including general and earmarked taxes (10% tax on mobile phone company turnover and a compulsory levy called the Special Solidarity Contribution), and social security contributions (employer–employee contributions).^31 32^

For countries that have already established publicly owned health insurance schemes, one consideration would be to repurpose them as strategic purchasers of healthcare services rather than revenue mobilisation agencies. Revenue mobilisation could be moved to the country’s tax agencies, accompanied by the reorientation of healthcare financing towards tax funding. Under such an arrangement, revenues for the health sector are collected through taxes (direct and indirect) and transferred to the purchasing agency to purchase services for the whole population. This is similar to the arrangement in Thailand where revenues are collected through tax and allocated to the National Health Securities Office which then purchases healthcare for the poor and the informal sector.^33^

Countries that have not set up, but are planning to set up, publicly owned health insurance schemes should reconsider this decision. Perhaps a more feasible path for them would be to strengthen their tax funding system by increasing funding allocation to the health sector, strengthen the supply-side capacity of public healthcare facilities and adopt strategic purchasing practices. This includes establishing independent healthcare purchasing authorities that can then enter into contracts with both public and private healthcare facilities for the provision of healthcare services. Ultimately, moving from voluntary contributions to tax funding will not only resolve the challenge of low coverage but will also contribute to tackling the prevalent inequalities characteristics of health insurance schemes in SSA.

## Data Availability

Data are available in a public, open access repository.
